# Ecotoxicological effects of carbon nanotubes and cellulose nanofibers in *Chlorella vulgaris*

**DOI:** 10.1186/1477-3155-12-15

**Published:** 2014-04-22

**Authors:** Michele M Pereira, Ludovic Mouton, Claude Yéprémian, Alain Couté, Joanne Lo, José M Marconcini, Luiz O Ladeira, Nádia RB Raposo, Humberto M Brandão, Roberta Brayner

**Affiliations:** 1 Nucleus of Analytical Identification and Quantification (NIQUA), Federal University of Juiz de Fora, 36036-900 Juiz de Fora, Brazil; 2 Interfaces, Traitements, Organisation et Dynamique des Systèmes (ITODYS), University of Paris Diderot, Sorbonne Paris Cité, 7086 Paris, France; 3 Muséum National d’Histoire Naturelle, Département Régulations, Développement et Diversité Moléculaire (RDDM), 75005 Paris, France; 4 Institut Jacques Monod, CNRS, University Paris Diderot, 75205 Paris, France; 5 Embrapa Instrumentation (CNPDIA), National Nanotechnology Laboratory for Agriculture (LNNA), 13560-970 São Carlos, Brazil; 6 Department of Physics, Federal University of Minas Gerais, 31270-901 Belo Horizonte, Brazil; 7 Embrapa Dairy Cattle (CNPGL), 36038-330 Juiz de Fora, Brazil

**Keywords:** Nanoparticle, Uptake, Nanotoxicity, Microalgae, Bioindicator

## Abstract

**Background:**

MWCNT and CNF are interesting NPs that possess great potential for applications in various fields such as water treatment, reinforcement materials and medical devices. However, the rapid dissemination of NPs can impact the environment and in the human health. Thus, the aim of this study was to evaluate the MWCNT and cotton CNF toxicological effects on freshwater green microalgae *Chlorella vulgaris.*

**Results:**

Exposure to MWCNT and cotton CNF led to reductions on algal growth and cell viability. NP exposure induced reactive oxygen species (ROS) production and a decreased of intracellular ATP levels. Addition of NPs further induced ultrastructural cell damage. MWCNTs penetrate the cell membrane and individual MWCNTs are seen in the cytoplasm while no evidence of cotton CNFs was found inside the cells. Cellular uptake of MWCNT was observed in algae cells cultured in BB medium, but cells cultured in Seine river water did not internalize MWCNTs.

**Conclusions:**

Under the conditions tested, such results confirmed that exposure to MWCNTs and to cotton CNFs affects cell viability and algal growth.

## Background

In recent years, many newly engineered nanomaterials are being developed due to the fast-growing area of nanotechnology*.* CNT and CNF are NPs that have received considerable attention. CNTs have unique characteristics, such as large contact surface, stability, flexibility, stiffness, strength, thermal and electrical conductivity. CNF has emerged as an attractive nanomaterial due to their hydrophilicity, flexibility, mechanical strength, broad chemical-modifying capacity, biodegradability aspect and low cost. Thus, CNTs and CNFs are noteworthy NPs, which encompass a number of potential applications, being used in water treatment, cosmetics, as well as reinforcement materials, biosensors and medical equipment [1-4].

Nevertheless, the rapid dissemination of NPs can cause an impact on the environment and on human health. So far, however, most nanomaterial-based publications are focused on the synthesis and development of new nanomaterials, and few studies have focused on NPs’ ecotoxicological impact. Some works have investigated the impact of CNTs on algal ecosystems [5-7]. Thus, at present, the knowledge on the ecotoxicological effects of CNTs is still limited, despite the large number of ongoing studies. Notably, in the case of cotton CNFs, no work, until now, has studied the potential cytotoxicity to microalgae cells and only one study suggested that cotton CNFs were genotoxic in plant cells [8]. Therefore, the ecotoxicological impact of CNTs and CNFs has to be determined. For this purpose, *Chlorella vulgaris* is a valuable bioindicator of potentially toxic elements and due to the ecological position of this organism at the base of the aquatic food chain and oxygen production. The objective of the current paper is to elucidate whether MWCNTs and cotton CNFs are toxic to *C. vulgaris* in BB medium or in natural water (Seine River).

This work provides a direct comparison of the impact of MWCNTs and cotton CNFs to *C. vulgaris*, either in BB culture medium or in Seine river water. To our knowledge, the interactions between MWCNTs or cotton CNFs and *C. vulgaris* in different types of growth medium have not been studied.

## Results and discussion

### Characterization of NPs and suspensions

**Figure 1:**
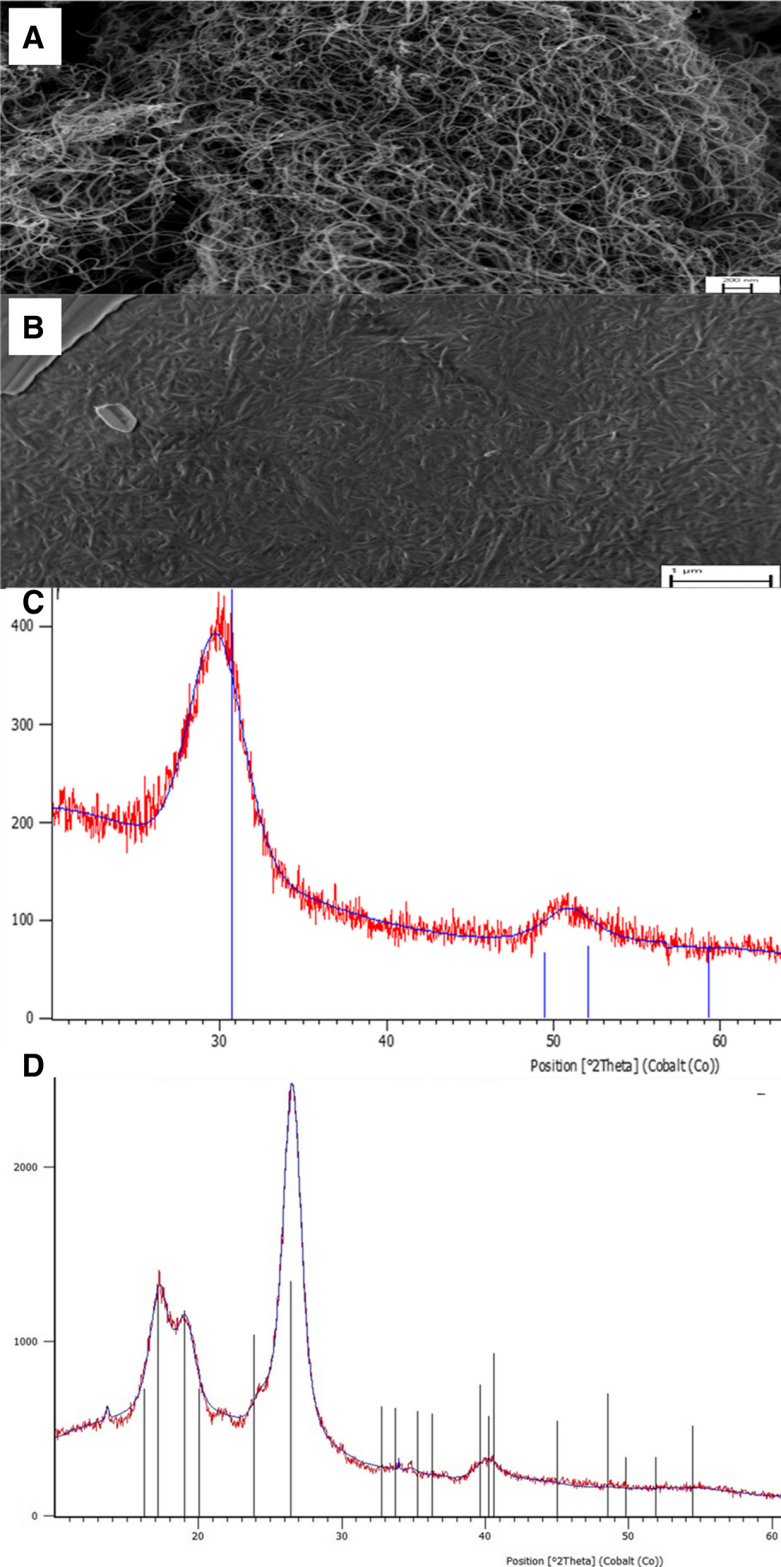
**Nanoparticles characterization.** SEM images of the Multi-walled carbon nanotubes (MWCNTs) **(A)** and cotton cellulose nanofibers (CNFs) **(B)**. X-ray diffraction patterns of the MWCNTs **(C)** and cotton CNFs **(D)**.

**Figure 2:**
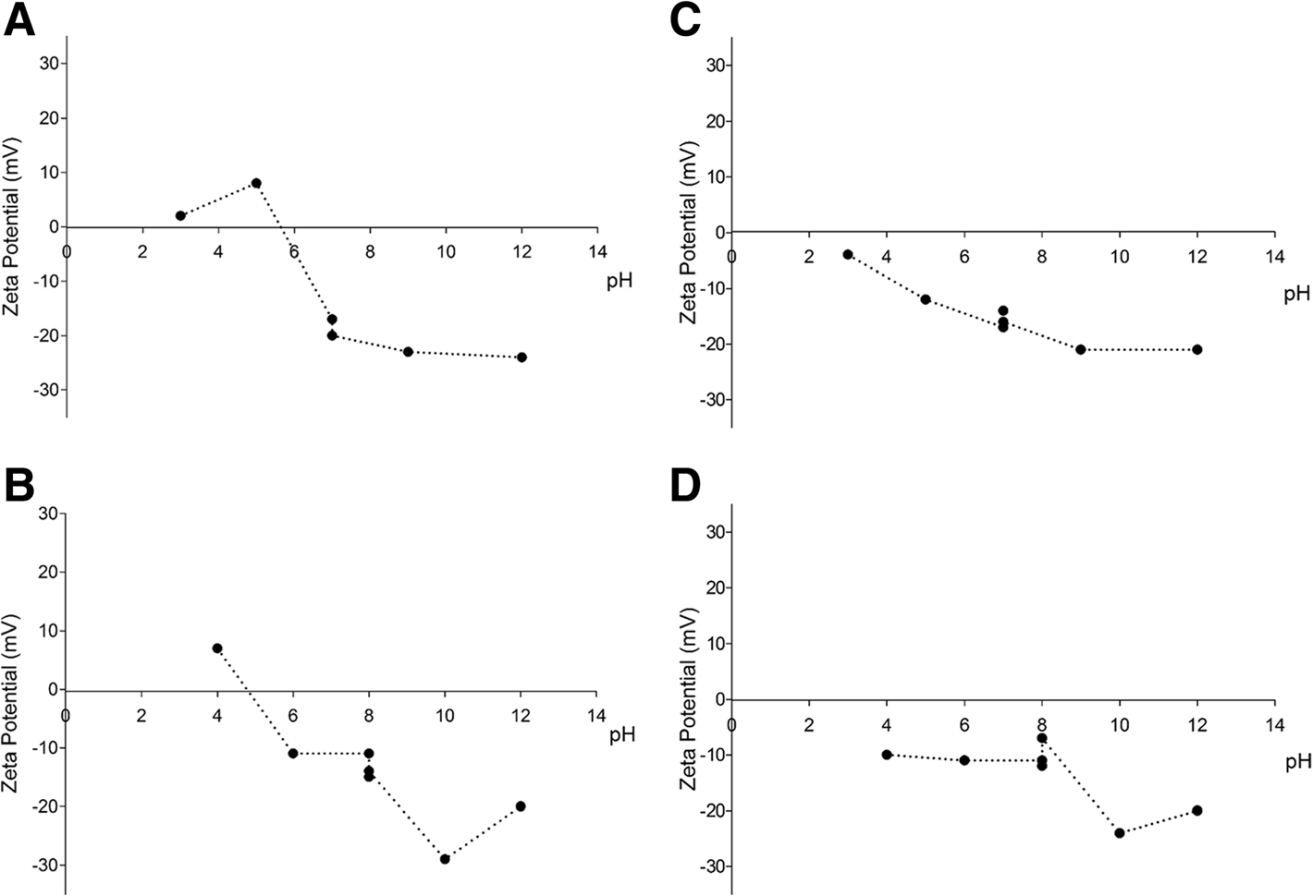
**Behavior of Zeta Potential of *Chlorella vulgaris *exposed to nanoparticles.***C. vulgaris* exposed to Multi-walled carbon nanotubes (MWCNT) in Bold’s basal (BB) culture medium **(A)** and Seine river water **(B)** at different pH. *C. vulgaris* exposed to cotton cellulose nanofibers (CNFs) in BB culture medium **(C)** or Seine river water **(D)** at different pH. The ZP decreases with increasing pH. Data are presented as mean from three independent experiments.

SEM images of the nanomaterials we used are presented in Figure 1A and B. The XRD patterns in Figure 1C and D indicate that both NPs have pure structural characteristics of MWCNT and cotton CNF materials. Before contact with the microorganisms, the ZP of MWCNT and CNF nanoparticles with varying pH of the media (BB and Seine river water) was measured after 30 minutes of contact between the nanoparticles and the pH solutions (Figure 2). The ZPC for MWCNT nanoparticles was observed at pH 4.0 and pH 4.8 for the BB medium (Figure 2A) and Seine river water solutions (Figure 2B), respectively. For the CNF nanoparticles, the ZPC is at a pH < 2 in both BB culture medium (Figure 2C) and in the Seine river water (Figure 2D).

Both MWCNT and CNF nanoparticles are negatively charged at neutral pH (7.0). *C. vulgaris* is also negatively charged at this pH. We expected no interactions between nanoparticles and *C. vulgaris.* This behavior was observed in Seine river water, on the other hand, the ZP was changed in BB medium due to the high ionic strength of this medium. CNF nanoparticles are most positively charged than MWCNT. For CNF materials the ZP changed in both media.

These results suggest that there are few cationic sites for adsorption of the negatively charged NPs. It is well known that positively charged NPs have more cellular uptake than negative NPs, due to the attractive electrostatic interactions with the cell membrane. However, anionic NPs bind to the cell surface on the form of clusters because of their repulsive interactions with the large negatively charged domains of the cell surface [9]. In addition, Patil et al. [10] showed the high cellular uptake of negatively charged nanoparticles and suggest that this is related to the non-specific process of NP adsorption at the positively charged sites on the cell-membrane. In fact, cytotoxicity assay and microscopy results showed interactions between NPs and algae cells.

### Effect of NPs on algae growth and viability

**Figure 3:**
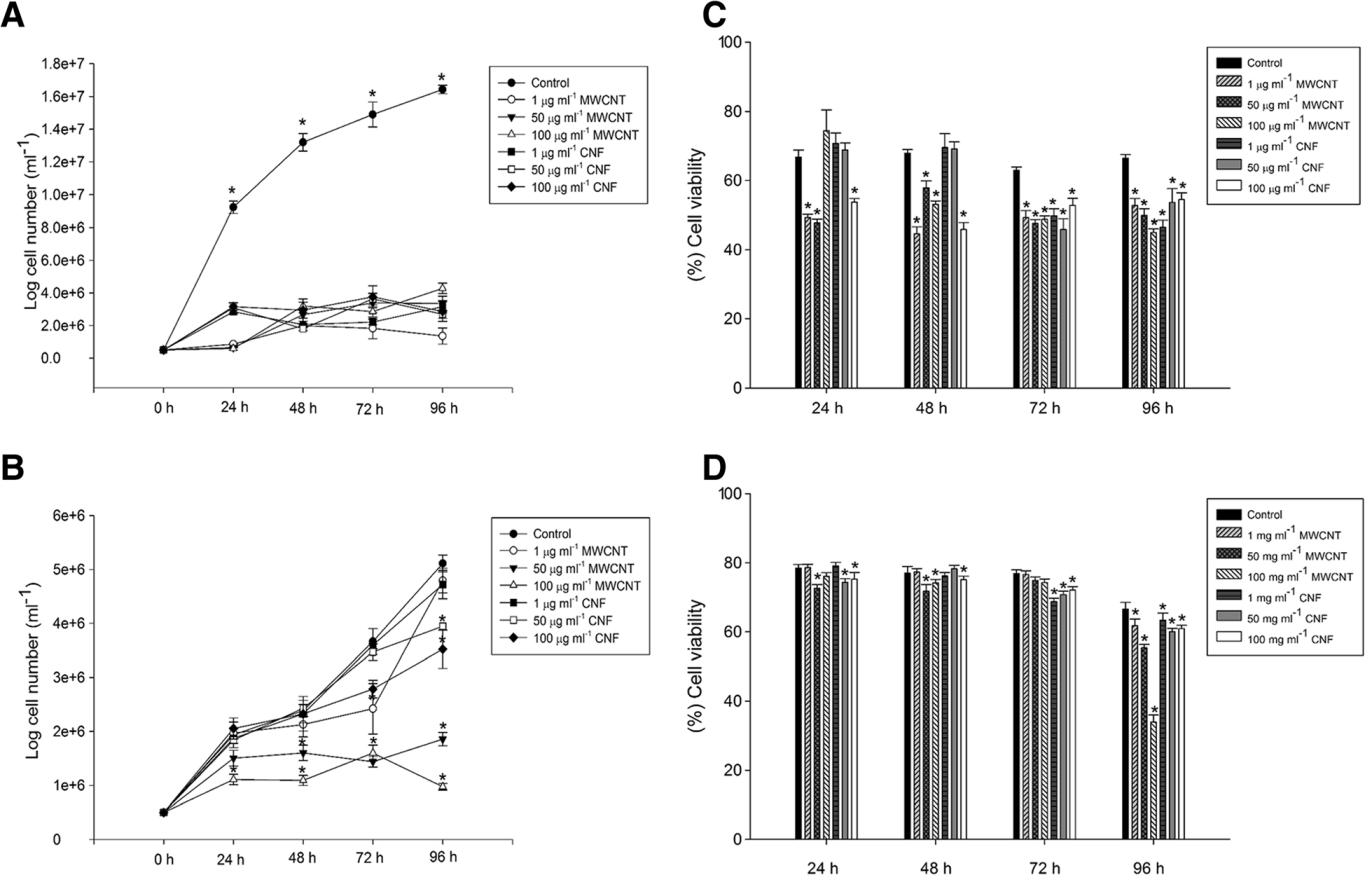
**Effect of nanoparticles on cell growth/cell viability of *Chlorella vulgaris*.** Cell growth of *C. vulgaris* after exposure to Multi-walled carbon nanotubes (MWCNTs) or cotton cellulose nanofibers (CNFs) at various incubation concentrations (1, 50 and 100 μg ml^−1^) and time points (24, 48, 72 and 96 hours) in Bold’s basal (BB) culture medium **(A)** and Seine river water **(B)**. Cell viability of *C. vulgaris* after exposure to MWCNTs or cotton CNFs at various incubation concentrations (1, 50 and 100 μg ml^−1^) and time points (24, 48, 72 and 96 hours) in BB culture medium **(C)** and Seine river water **(D)**. Data are presented as mean ± SEM from three independent experiments. Groups significantly different from the control group (by ANOVA followed by Student–Newman–Keuls’ test) are shown by *p < 0.05.

**Figure 4:**
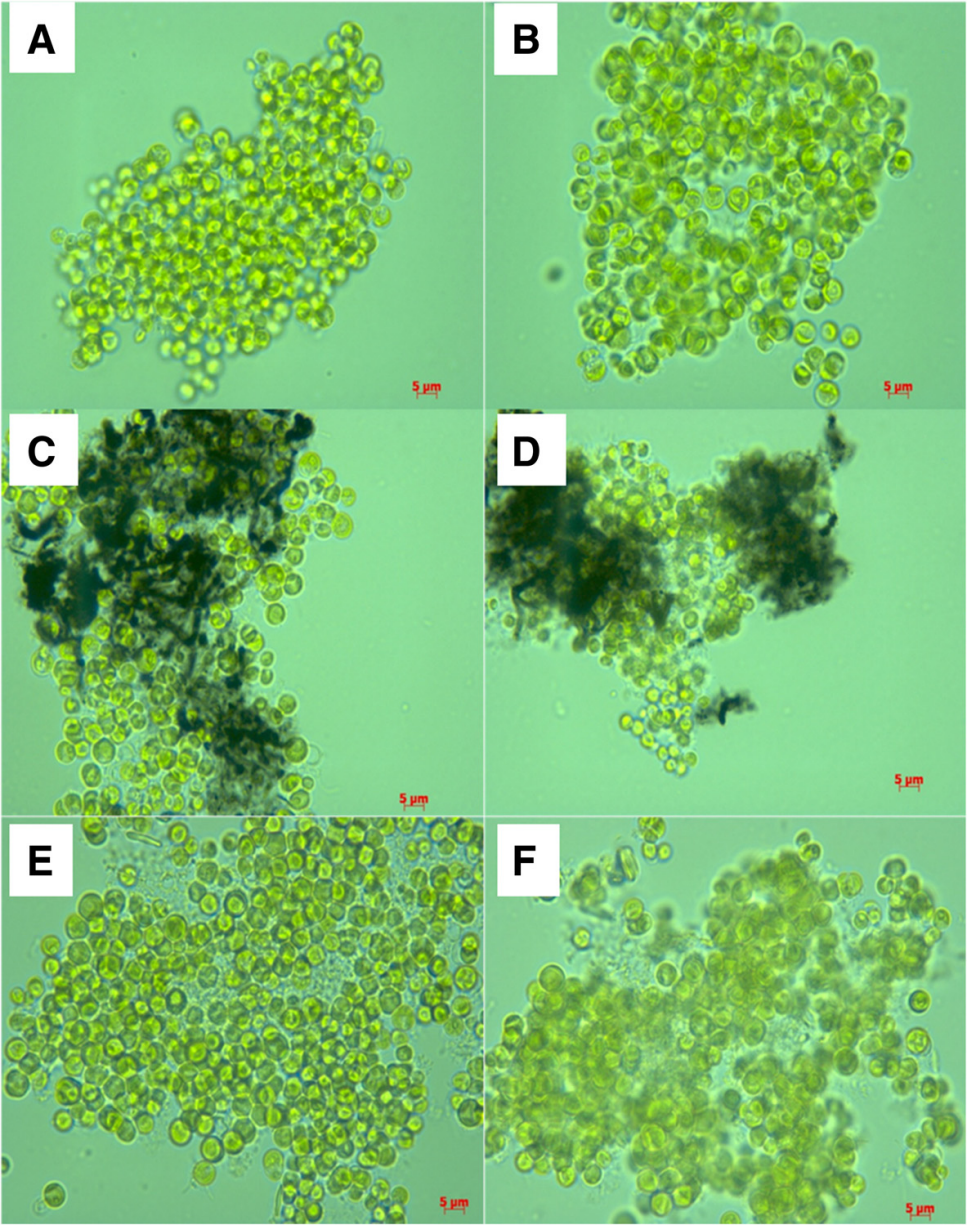
**Optical micrographs of *Chlorella vulgaris *treated with different nanoparticles at 100 μg ml**^**−1 **^**for 24 hours.** Control Bold’s basal (BB) culture medium **(A)**, Control Seine river water **(B)**, Multi-walled carbon nanotubes (MWCNT) in BB culture medium **(C)**, MWCNT in Seine river water **(D)**, cotton cellulose nanofibers (CNFs) in BB culture medium **(E)** and CNF in Seine river water **(F)**. Note nanoparticle aggregates in *C. vulgaris* cells. Bars, 5 μm. Magnification 400 × .

The effect of NPs on the viability of *C. vulgaris* was assessed by direct cell counting. Figure 3A shows the toxic effect of NPs on *C. vulgaris* cultured in BB medium as a function of concentrations and exposure times. After 24 hours of exposure, the cell numbers were changed (P < 0.001). Interestingly, NP exposure led to a decreased in the number of cells, in a non-dose-dependent manner, and for both NPs, the inhibition of algal growth rate occurred at the concentration of 1 μg ml^−1^. Such findings are in agreement with previous studies, showing that the CNTs reduced the algal growth of *C. vulgaris*[5,7,11]. Microscopy analyses showed aggregation between NPs and microalgae (Figure 4). Previous work reported that the proximity of algal cells clogged inside CNT agglomerates lead to different growth conditions [12]. Such behavior can disrupt the supply of sufficient nutrients, which is a crucial factor to the microalgae growth [13]. Additionally, Sargent et al*.*[14] demonstrated disruption in the mitotic spindle by SWCNTs. Thus, in the present study, the growth inhibition caused by these NPs was most likely the result of insufficient illumination and nutrient availability of algal cells in agglomerates of NPs. In Seine river water, a decrease in algal growth was only observed after 24 hours of exposure (100 μg ml^−1^ MWCNT) (P = 0.022) (Figure 3B). This behavior may be due to the presence of natural polymers in Seine river water such as fulvic and humic acids that can adsorb on the particle surface.

In the present study, the impact of NPs on algae membrane integrity was assessed with the Trypan Blue assay. Exposure of *C. vulgaris* cells to MWCNTs or cotton CNFs led to significant reductions in algal viability, depending on the dosage and exposure time (Figure 3C and D). From the results, it could be seen that MWCNTs in BB medium caused a reduction of cell viability at all concentrations tested (45.50 − 69.83% relative to controls; P < 0.001). However, for cotton CNFs (1 and 50 μg ml^−1^), the toxicity (50.50 and 48.83%, respectively; P < 0.001) was only observed over 72 hours of exposure (Figure 3C).

On the other hand, cells that were incubated in Seine river water during exposure to MWCNTs did not show a decrease in viability from 1 μg ml^−1^ to 72 hours, but at 96 h, a reduction in cell viability to 61.70% (P = 0.038) was observed, when compared to control 66.56% (Figure 3D). A particularly drastic decrease in cell viability (P < 0.001) was observed at high concentrations (50 and 100 μg ml^−1^) of MWCNTs (55.33% and 33.95%, respectively; Figure 3D). Such results are consistent with other CNT cell viability studies, albeit in different cell types [5,14,15].

For cotton CNFs in the Seine river water, all concentrations were toxic, especially after 72 hours of exposure (Figure 3D). Recent reports indicated the toxicity of cotton CNFs on mammalian and plant cells. Clift et al. [16] showed low *in vitro* cytotoxicity of cotton CNFs in human lung cells. Previous work in our laboratory showed that high concentrations (2000 and 5000 μg ml^−1^) of cotton CNFs cause a decrease in cell viability in bovine fibroblasts Pereira et al. [17]. In particular, cotton CNFs were reported to be genotoxic in plant cells [8]. Our results are in agreement with these previous studies.

### Photosynthetic activity

**Figure 5:**
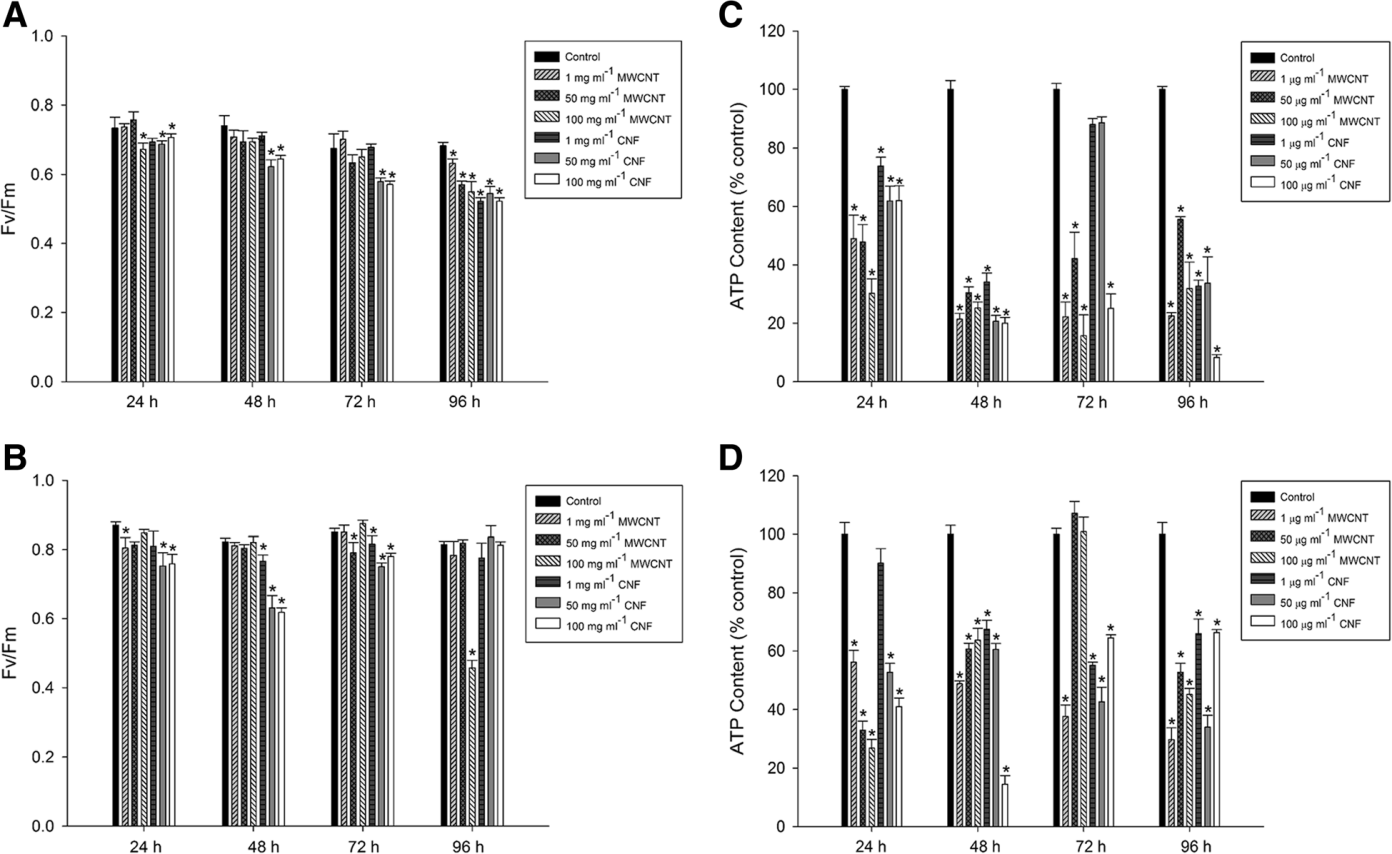
**Influence of nanoparticles to activity of photosynthetic apparatus (Fv/Fm) and ATP levels of *Chlorella vulgaris.*** Maximum quantum efficiency of the photosystem II (FV/Fm) of *C. vulgaris* cultured under different concentrations (1, 50 and 100 μg ml^−1^) of Multi-walled carbon nanotubes (MWCNTs) or cotton cellulose nanofibers (CNFs) at various time points (24, 48, 72 and 96 hours). Bold’s basal (BB) culture medium **(A)** and Seine river water **(B)**. ATP levels of *C. vulgaris* cells following at 24, 48, 72 and 96 hours coincubation with either MWCNTs or cotton CNFs (1, 50 or 100 μg mL^−1^) in BB culture medium **(C)** and Seine river water **(D)**. Data are presented as mean ± SEM from three independent experiments. Groups significantly different from the control group (by ANOVA followed by Student–Newman–Keuls’ test) are shown by *p < 0.05.

The photosynthetic activity of *C. vulgaris* after addition of MWCNTs or cotton CNFs was measured using a PAM fluorimeter (Figure 5A and B). For BB medium, 1 and 50 μg ml^−1^ MWCNTs or 1 μg ml^−1^ cotton CNFs did not influence the photosynthetic activity of *C. vulgaris* after 72 hours exposure (P > 0.05). However, the photosynthetic activity decreases (0.673 ± 0.03; P = 0.004) for the cells exposed to 100 μg m^−1^ MWCNs after 24 hours exposure (Figure 5A). After 96 hours, for MWCNT nanoparticles, a decrease of the photosynthetic activity at all concentrations (P < 0.05) was observed. For cotton CNFs the Fv/Fm decrease was significant (0.522 ± 0.01; P < 0.001) for 1 μg ml^−1^, only after 96 hours of exposure. On the other hand, the photosynthetic activity decreases with time after contact with 50 and 100 μg ml^−1^ concentrations (P < 0.05, Figure 5A).

In the case of Seine river water (Figure 5B) no photosynthetic activity variation was observed (P > 0.05) after contact with 1 μg ml^−1^ MWCNT after 48 and 96 hours as well as after contact with 50 μg ml^−1^ after 24, 48 and 96 hours and after contact with 100 μg ml^−1^ between 24 and 72 hours. However, for 1 μg ml^−1^ MWCNT after 24 hours (0.805 ± 0.05, P = 0.04), 50 μg ml^−1^ MWCNT after 72 hours (0.791 ± 0.05, P < 0.001) and 100 μg ml^−1^ after 96 hours (0.458 ± 0.03, P < 0.001), the photosynthetic activities decreased significantly. No changes occurred in cells exposed to 1 μg ml^−1^ cotton CNF after 24 hours and 50 μg ml^−1^ cotton CNFs after 96 hours (P > 0.05). However, for all other conditions photosynthetic activity alteration was observed (P < 0.05; Figure 5B).

The present findings seem to be consistent with other studies which found that algal photosynthetic activity was also suppressed at nano-Ag [18], ZnO [19] and nano-TiO_2_[20]. However, Schwab et al. [10] demonstrated that the photosynthetic yield of *C. vulgaris* remained unchanged, even at concentrations up to 40 mg pristine or oxidized CNT/L. This inconsistency may be due to the chemical functionalization of the CNT. In the current study, we used non-functionalized MWCNTs. Several studies have revealed that CNT surface functionalization may alter the toxicity response [21-23].

Gao et al. [24] found that nanomaterial toxicity after contact with photosynthetic organisms is also exhibited by reductions in the photochemical efficiency of the PSII. A decrease in the photosynthetic activity may be caused by a defect in the quantum yield of PSII itself, such as non-photochemical quenching [25]. It is possible, therefore, that long-term exposure or high concentrations of NPs affect the photosynthetic rate in *C. vulgaris* via alterations in the PSII photochemical efficiency. Further research should be done to investigate this. Microscopy analysis showed interaction between NPs and microalgae (Figure 4C-F). It can thus be suggested that the accumulation of NPs on the surface of *C. vulgaris* cell walls may inhibit photosynthetic activity because of shading effects, i.e., reduced light availability. In addition, the primary cause of the observed photosynthetic inhibition by NPs in green microalga could be an excessive level of ROS formation [25]. To further investigate this, we examined whether the MWCNTs and the cotton CNFs have cytotoxic impact by altering the intracellular oxidative status.

### Effect of NPs on SOD activity

The activity of the antioxidant enzyme superoxide dismutase (SOD) was determined in *C. vulgaris* after exposure to NPs. SOD activity increased (P < 0.05) in cells exposed to MWCNTs and cotton CNFs in BB culture medium and remained higher than the controls at all-times except for 100 μg ml^−1^ after 96 hours (see Additional file 1: Table S1). In Seine river water, an increase of SOD activity was observed after 24 hours (P < 0.05; see Additional file 1: Table S1). These results are consistent with previous studies, which have shown that the CNT treatment can induce significant ROS production and influence cell viability [26-28]. Interestingly, no differences (P > 0.05) were found in cells exposed to 50 μg ml^−1^ cotton CNF after 48, 72 and 96 hours and 100 μg ml^−1^ after both 48 and 96 hours (see Additional file 1: Table S1). In addition, no differences (P > 0.05) were found between 50 μg ml^−1^ MWCNT and the control after 96 hours (see Additional file 1: Table S1).

Cheng et al. [29] showed that the ROS generation was involved in the activation of the mitochondria-dependent apoptotic pathway in cells exposed to CNTs. These findings further support the idea that nanoparticle-induced ROS production in cells can lead to cell death. In the present study, a decrease in cell viability was observed when algae cells were exposed to MWCNTs and cotton CNFs. On the other hand, Meng et al. [30] suggested that ROS were not widely generated by carboxylated MWCNTs incubation. As previously discussed, the functionalization of CNTs can alter their cellular interaction pathways. The potential impact of cotton CNFs on cell oxidative stress is little known. In a recent study, exposure to cotton CNFs resulted in an increase of oxidative stress response gene expression in mammalian fibroblast [17]. This finding corroborates the results in the present study, which showed oxidative stress on microalgae exposed to cotton CNFs (see Additional file 1: Table S1).

SOD is one of the most important antioxidative enzymes, which catalyzes the superoxide dismutation (O_2_^−^) into oxygen and hydrogen peroxide. It plays an important role in the protection of cells against ROS by lowering the steady state of superoxide anions. The increased activity of SOD in cells after contact with MWCNTs and cotton CNFs suggests a possible survival mechanism for *C. vulgaris*, in order to reduce possible cytotoxic effects such as cell death. However, data from cell viability showed that under some exposure conditions the cellular antioxidant system may not be able to prevent cell death induced by NPs. Thus, the production of ROS is one of the key factors contributing to the toxicity of nanomaterials in freshwater green microalgae.

In the Seine river water, only at 1 μg ml^−1^ MWCNT concentration after 96 hours, a decrease in SOD activity was observed, when compared to the control (see Additional file 1: Table S1). The exact cause of the decrease in SOD activity in cells exposed to 1 μg ml^−1^ MWCNTs in Seine river water is not known. It has been suggested that the oxidative stress and the accumulation of hydrogen peroxide, which irreversibly inactivates SOD, might possibly disturb SOD synthesis by damaging the mitochondrial function [31]. Another possible explanation is that the impairment in the antioxidant defense system weakens ROS detoxification, which exacerbates cell death when such cells are exposed to an acute oxidative challenge [32]. Hence, it could be conceivably hypothesized that *C. vulgaris*, under certain culture conditions, may be more vulnerable to oxidative stress as a result of a greater oxidative burden, or, alternatively, lower antioxidant protection. Since the formation of ROS by MWCNTs and cotton CNFs is unclear, the mechanism of ROS formation by these NPs needs further investigation.

### Effect of NPs on ATP production in microalgae cells

Since a cellular redox change may decrease the energy production in the form of ATP from mitochondria, we examined intracellular ATP levels. Figure 5 shows the decline in ATP levels in cells after exposure to NPs. The ATP levels after contact with both MWCNT and cotton CNF decreased, and after 48 hours they were significantly lower than the levels in the control cells (Figure 5C and D). However, ATP levels were not changed (P > 0.05) after 72 hours of exposure to 1 and 50 μg ml^−1^ cotton CNFs (BB culture medium) or after 24 hours of exposure to 1 μg ml^−1^ cotton CNF and after 72 to 50 and 100 μg ml^−1^ (in the Seine river water) (Figure 5C and D). Little is known about the potential effects of NPs on mitochondria. Some studies showed that particle-ultra-fine carbon black and nanoscale zerovalent iron caused a decrease in ATP levels [33,34]. In addition, SWCNTs decrease mitochondrial membrane potential, inducing the formation of ROS on neuronal cells [35].

Mitochondria are responsible for an efficient coupling of cellular respiration to ATP production. Thus, mitochondrial dysfunction can contribute to cell death by reducing ATP production, increasing ROS production and releasing regulatory death [36]. Additionally, Fariss et al. [37] reported that ROS cause damage to the mitochondrial genome, impairing its activity*.* ATP is a universal energy unit in all living cells, and a decline in ATP levels is indicative of loss in mitochondrial function. In the current study, the variation of intracellular ATP levels after contact with MWCNT and cotton CNF particles compared to untreated cells, suggest that both MWCNTs and cotton CNFs impair the energetic metabolism of *C. vulgaris.*

### Microscopic study of microalgal cells exposed to NPs

In order to further explore the cellular mechanisms of the observed NP toxicity, we used optical, SEM and TEM microscopies of the *C. vulgaris* cells exposed to MWCNTs and cotton CNFs in both BB culture medium and Seine river water. The optical microphotographs for *C. vulgaris* exposed to NPs at the highest concentration (100 μg ml^−1^) show the formation of particle aggregates on algal cells after 24 hours (Figure 4C-F). Figure 4C, D and F demonstrate that the majority of cells are completely trapped inside the shell of NPs. Under these circumstances, cells probably undergo stress due to the lack of essential nutrients and energy. To corroborate our results, it was suggested by Rodea-Palomes et al. [38] that the *Anabaena* cells trapped inside the nanoparticle shell, as well as the transport of nutrients and metabolites across the cell wall and membrane could be affected, leading to cell death. The aggregation of NPs depends on particle concentrations, pH, zeta potential and the characteristics of the aqueous media [39]. It is well known that those particles with more positive ZP than +30 mV or more negative ZP than −30 mV are normally considered to be stable [23]. Thus, ZP is a critical parameter, which determines nanoparticle stability or aggregation in dispersion. In the present study, all absolute ZP values were lower than 30 mV (Figure 2). Our results demonstrate that NPs in both BB culture medium and Seine river water are unstable solutions. This finding may be explained by the fact that both neutral pH in the cell medium (pH 7.4) and the high ionic strength led to a higher degree of NP agglomeration [15]*.* In addition, an essential property governing the behavior of NP suspension in aqueous media is their tendency to form aggregates [38]. The affinity of microalgal cells to MWCNTs and cotton CNFs suggests that there must be a specific interaction between NPs and the *C. vulgaris* surface, strong enough to override the electrostatic repulsion of the negatively charged NPs and microalgae (observed in ZP, Figure 2).

Several factors govern the effect of NPs on microalgal growth, among them we can cite flocculation, light and nutrient availability [40]. A previous study revealed that growth inhibition was highly correlated with the shading of CNTs and the agglomeration of algal cells [7]. MWCNTs, at high concentrations, were shown to impair cell viability and this effect seems to be due to the strong tendency of CNTs to agglomerate [41]. In addition, Clément et al*.*[42] showed that aggregates reduce the fluorescence of algal cells, affecting toxicity. Therefore, the important problem encountered in the investigation of CNT toxicity is the tendency to aggregate [43]. Here, the results from Trypan blue exclusion assay and optical microscopy suggest that the formation of NP aggregates might alter the cellular acquisition of essential nutrients by cells and decrease cell viability.

**Figure 6:**
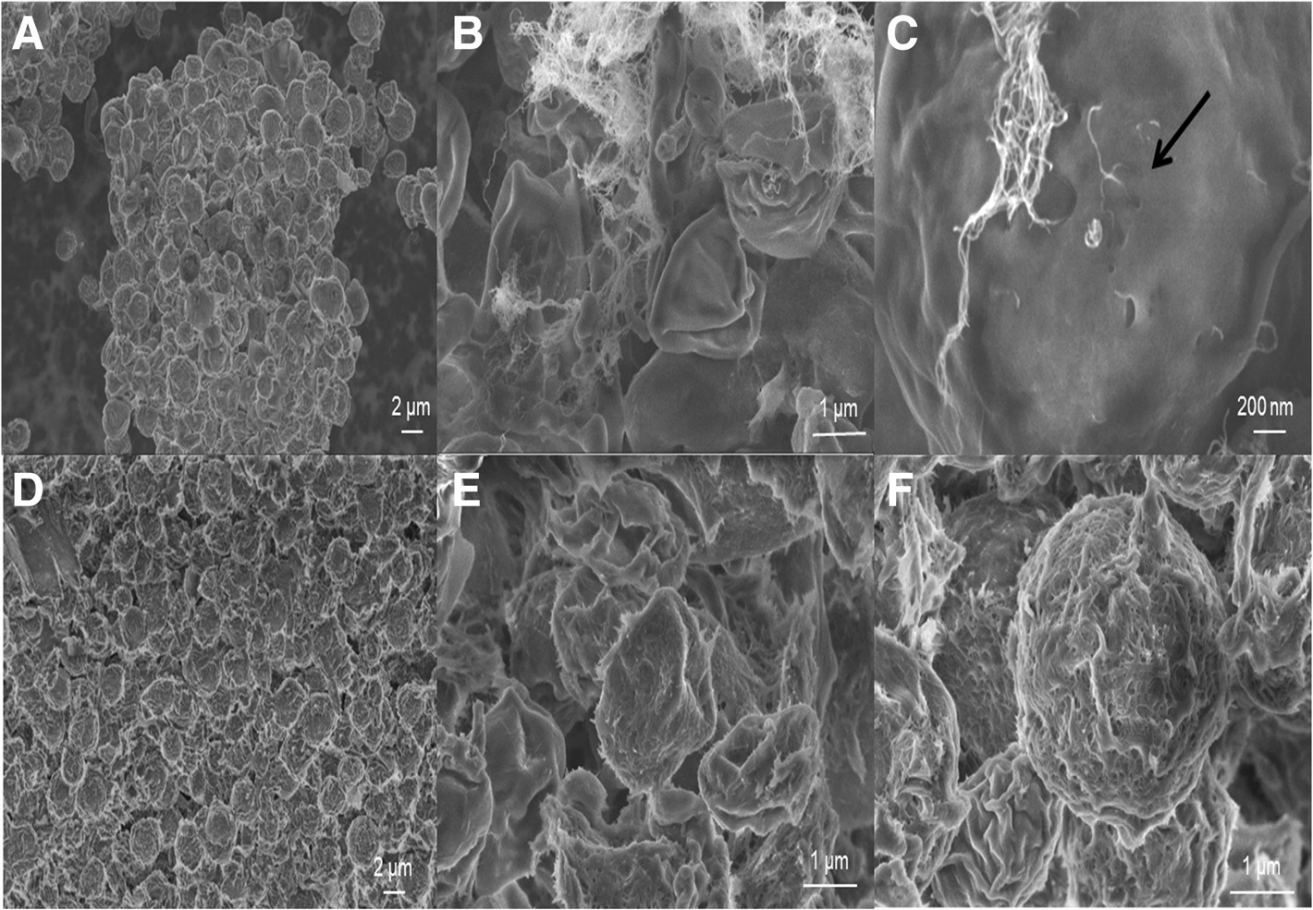
**SEM micrographs of *Chlorella vulgaris *exposed to different nanoparticles at 100 μg ml**^**−1 **^**for 48 hours.** Control cell **(A)**, *C. vulgaris* cells in close contact to Multi-walled carbon nanotubes aggregates **(B-C)**, the black arrows indicate highly damaged cell **(C)**. *C. vulgaris* cells coated with cotton cellulose nanofibers **(D-F)**. Detail of highly damaged *C. vulgaris* cell **(E)**.

In order to evaluate morphological, cellular ultrastructure changes and interaction between NPs and *C. vulgaris*, we analyzed microalgae cells by SEM and TEM after 48 hours of contact with MWCNT and cotton CNF (100 μg ml^−1^). In the control group (Figure 6A), the cell structure is intact, and the shape of the *C. vulgaris* appears round. However, cell shrinkage was noted on the cells treated with both MWCNTs and cotton CNFs (Figure 6B and E). Figure 6F shows gum-like cellulose surrounding the microalgae. Previous studies have shown that the interaction between NPs and phytoplankton produced EPS [19,44]. The increased production of EPS was found to be a general response to the presence of pollutants [45]. Brayner et al. [19] suggested that the polysaccharides produced by *Anabaena flos-aquae* avoid particle internalization. On the other hand, here the production of EPS in microalgae may have increased the adsorption of NPs on the cell surface. Cellulose is made of long sugar molecule chains. During hydrolysis, such chains are broken down into simple sugars. In the present study, it is possible to hypothesize that cotton CNF chains have been broken down producing a glue gel-like water suspension. Therefore, a strong interaction between cotton CNFs and the EPS produced by *C. vulgaris* is possible*.* The adsorption of the NPs to the cell wall may impair gas exchange and transport nutrients across the plasma membrane. Thus, such cotton CNF adsorption surface is most probably the cause of the observed reduction in the photosynthetic activity (Figure 5A and B).

**Figure 7:**
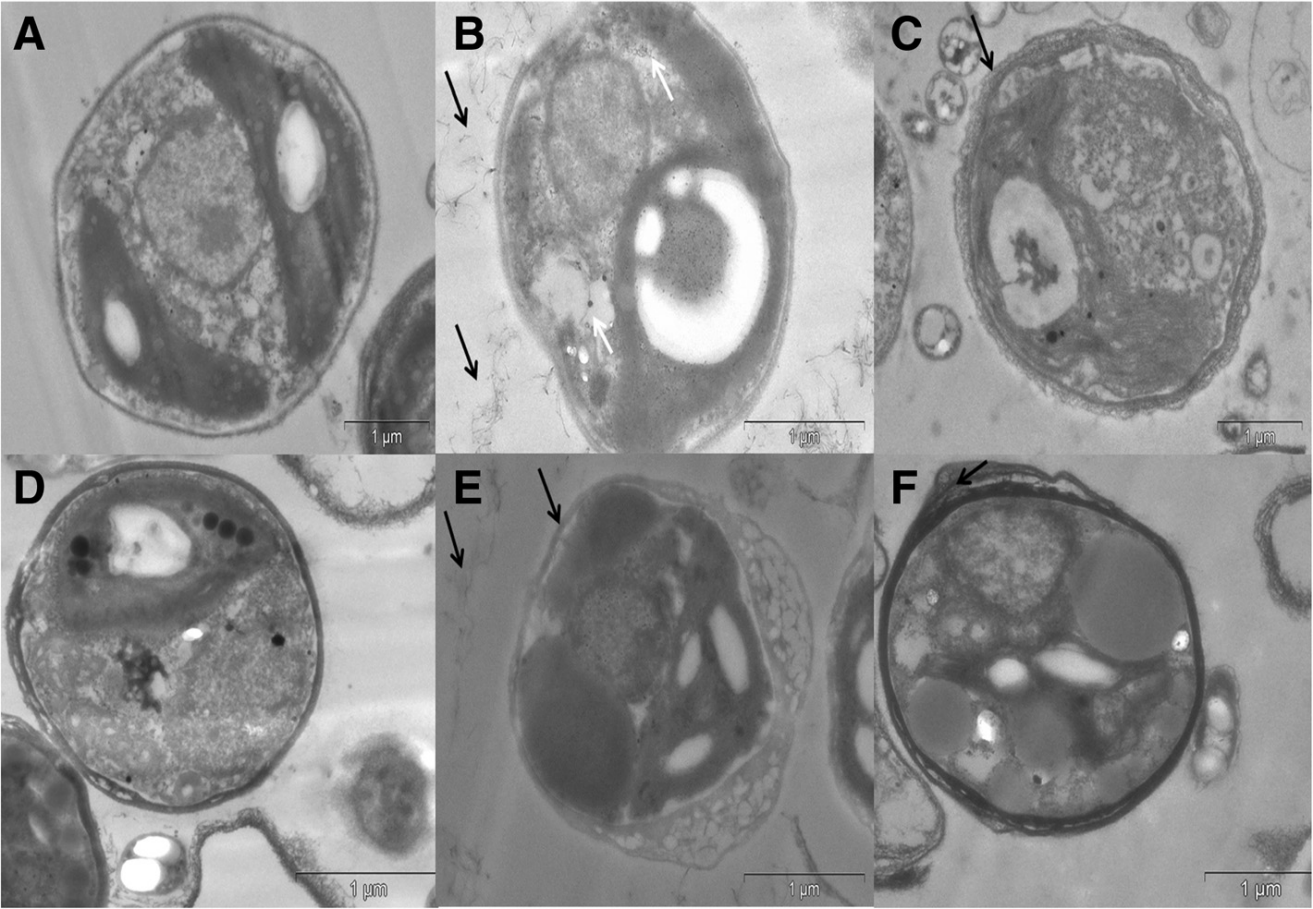
**TEM results of *Chlorella vulgaris *exposed to different nanoparticles at 100 μg ml**^**−1 **^**for 48 hours.***C. vulgaris* cell cultured with Bold’s basal (BB) culture medium only **(A)**, *C. vulgaris* cell cultured with Multi-walled carbon nanotubes (MWCNTs) in BB culture medium **(B)**, *C. vulgaris* cell cultured with cotton cellulose nanofibers (CNFs) in BB culture medium **(C)**; *C. vulgaris* cell cultured with Seine river water only **(D)**, *C. vulgaris* cell cultured with MWCNT in Seine river water **(E)**, *C. vulgaris* cell cultured with cotton CNFs in Seine river water **(F)**. MWCNTs (small black tubes) are inside the cell **(B)** while there is no evidence of cotton CNFs was found inside the cells. Black arrows: extracellular MWCNTs or cotton CNF; white arrows: intracellular MWCNTs.

MWCNTs are commonly associated with the plasmatic membrane (Figure 6C), suggesting that CNTs could cross the cell wall and the plasma membrane and then enter into the cells. In fact, stained ultrathin sections from MWCNT-treated cells revealed the presence of CNTs in the cytoplasm (Figure 7B) while no evidence of CNFs was found inside the cells (Figure 7C). This result may be explained by the fact that the cellular uptake pathway of the NPs depends on the particle physico-chemical properties and the surface features. The aspect regarding high ratio, high stiffness and buckle flexing capabilities of MWCNT may have facilitated its crossing cellular barriers. *C. vulgaris* possesses cell walls which constitute a primary site for interaction and formation of a barrier against the entrance of NPs into their cells. In the present study, only free MWCNTs were introduced to the cells (Figure 7B). This finding corroborates the ideas of Wei et al. [46] who suggested that only NPs and NP aggregates with a smaller size are expected to pass through the cell walls and reach the plasma membranes.

Interestingly, cells in Seine River water did not show internalization of MWCNTs (Figure 7E). A possible explanation for this might be that the culture system type (BB culture medium or the River seine water) affects cellular uptake of MWCNTs in *C. vulgaris* cells. Recently, *in vitro* studies have shown that the culture medium composition may affect the proteins that are expressed on the cytoplasmic membrane, which may influence the internalization of MWCNTs [47]. In addition, in Seine River water, the interactions of natural polymers with MWCNT may prevent the internalization of this NP into *C. vulgaris* cells. Further research should be done to investigate the cellular uptake of MWCNTs in microalgae cells in different medium composition.

The observed adsorption of both MWCNTs and cotton CNFs to the cell surface may result in the disruption of the cell wall and membrane. Such loss in cell membrane integrity may lead to cell death. TEM images of microalgae exposed to MWCNTs both in BB culture medium and in Seine river water show damage to cell membranes (Figure 7B and E). The micrograph shows a cell, which has partly lost its cell wall and membrane. It is therefore likely that membrane damages lead to the release of cytoplasmic elements. Only dead cells, with disrupted plasma membrane, will be labelled by Trypan blue dyes. Thus, in this study, data from Trypan blue assay corroborate the microscopy findings.

It is not clear whether the internalization of particles is relevant to the induction of intracellular effects or the toxicity may be due to the adsorption of NPs on the cellular membrane [38]. In the present study, two hypotheses are possible: cotton CNFs anchor to the algal cell surface and internalization of the MWCNTs induces membrane rupture, morphologic alterations and toxic effects*.*

## Conclusions

Under the conditions tested, such results confirmed that exposure to MWCNTs and to cotton CNFs affects cell viability and algal growth. The toxicity of NPs on *C. vulgaris* has several causes. NPs appear to affect algae growth and cause cell death by inducing oxidation, disturbing ATP production, decreasing photosynthetic activity and physical stress. Cellular uptake of MWCNTs was observed in algae cells cultured in BB culture medium, but cells cultured in Seine river water did not internalize MWCNTs. Together, such factors *might have* contributed to the decline in culture algae exposed to NPs.

## Methods

### Preparation and dispersion of nanoparticles

The MWCNTs (diameters of about 20–40 nm and lengths of 40–60 μm) were synthesized by means of a floating catalytic chemical vapor deposition process using ferrocene and ethylene as the transition metal and carbon precursors, respectively. After synthesis, the MWCNTs were submitted to a simple purification process, by means of which they were washed and filtered several times with isopropyl alcohol in a Millipore filtration system, in order to remove any non-reacted ferrocene and other carbon impurities. After the cleaning process, the MWCNTs were dried at 80°C for 12 hours. The CNFs (diameters of about 6–18 nm and lengths of 85–225 μm) were prepared from 5 g of cotton fibers which were dispersed in 100 ml of 6.5 M sulfuric acid at 45°C and vigorously stirred for 75 minutes. After that, 500 ml of cold distilled water was added to stop the reaction. The sulfuric acid was partially removed from the resulting suspension by centrifugation at 8,000 × g for 15 minutes. The non-reactive sulfate groups were removed by centrifugation followed by dialysis. Then, the fibers were resuspended and dialyzed against tap water with a tubing cellulose membrane (76 mm, D9402-Sigma) until the pH reached 6–7. The resulting suspension was sonicated (Branson 450 sonifier, Branson Ultrasonics, Danbury, USA) for 5 minutes (in ice bath) and stored in a refrigerator. Stock solutions of the nanomaterials (10 mg ml^−1^) were prepared in BB culture medium or water from the Seine river, filtered by sonication in an ultrasonic bath during 20 minutes at 20 W.

### X-ray diffraction (XRD)

XRD patterns were recorded using an X’Pert PRO (PANalytical) diffractometer with Co Kα radiation. The diffractometer was calibrated using a standard Si sample. The samples were placed on a Si holder (absence of Si peaks).

### Zeta potential (ZP)

Zeta potential of NPs in BB culture medium or filtered Seine river water (0.22 μm) was determined using a Malvern zetasizer Nano ZS, at the same experimental conditions as used for the growth test. Three replicates per treatment were measured at 0, 24, 48, 72, and 96 hours. NP suspensions with and without algae, at different pHs, were compared. The zeta potential was plotted *vs.* pH and the value where the zeta potential equaled zero was taken to be the PZC.

### Scanning electron microscopy (SEM)

The samples were imaged by a Zeiss Supra 40 scanning electron microscope equipped with an in-lens detector. For imaging, low excitation voltage (2.5 kV) and a small working distance (6 mm) were used. Under these experimental conditions, charging effects were minimal, and therefore, it was not necessary to metalize the samples, so that true sample features were not masked.

### Culture conditions

*C. vulgaris* was grown in a 250 ml Erlenmeyer flask, with 0.22 μm vented plug seal cap, in sterile BB culture medium at pH 7.4 (adjusted with 1 M NaOH solution) or in Seine river water sterilized by 0.22 μm filtration at a controlled temperature of 20.0 ± 0.5°C and luminosity of 50–80 μmol m^−2^ s^−1^ photosynthetic photon flux. Appropriate concentrations of each nanomaterial stock solution were added to a microalgal culture in the exponential growth phase and incubated for 24, 48, 72 and 96 hours.

### Viability assay

The Cellometer Auto X4 simultaneously calculates cell concentration and % viability for cultured cells stained with Trypan blue. Trypan blue is a vital stain used to selectively color dead tissues or cells blue. It is a diazo dye. Live cells or tissues with intact cell membranes are not colored. Since cells are very selective in the compounds that pass through the membrane, in a viable cell, Trypan blue is not absorbed; however, it traverses the membrane in a dead cell. Hence, dead cells are shown as a distinctive blue color under a photonic microscope.

### PSII fluorescence measurements

The photosynthetic activity of microalgae was measured using the PAM method with a Handy PEA (Hansatech instruments) fluorometer. This method uses the saturation pulse method, in which a phytoplankton sample is subjected to a short beam of light that saturates the PSII reaction centers of the active chlorophyll molecules. This process suppresses photochemical quenching, which might otherwise reduce the maximum fluorescence yield. A ratio of variable-over-maximal fluorescence (Fv/Fm) can then be calculated which approximates the potential quantum yield of PSII.

### Superoxide dismutase (SOD) assay

The SOD activity was spectrophotometrically determined using the SOD kit (19160) purchased from Sigma-Aldrich (Chemie GmbH, Germany), following the instructions in the kit. After incubating the plate at 37°C for 20 minutes, absorbance was read at 450 nm using a microplate reader.

### ATP content bioluminescent assay

Intracellular levels of ATP were quantified with the ATP bioluminescent assay (Sigma) according to the manufacturer’s recommendations. Relative luminescent units were detected with an Envision Multilabel Plate Reader (Perkin-Elmer, Massachusetts, MA, USA).

### Optical microscopy

Interaction of NPs with *C. vulgaris* cells was studied by optical microscopy (Zeiss Primo Star microscope) after 24 hours of exposure to MWCNTs or cotton CNFs (100 μg ml^−1^).

### Transmission electron microscopy (TEM) and scanning electron microscopy (SEM)

After 48 hours exposure to MWCNTs or cotton CNFs (100 μg ml^−1^), the microalgae were fixed with a mixture containing 2.5% glutaraldehyde, 0.5% osmium tetroxide and 1.0% picric acid in a phosphate Sörengen buffer (0.1 M, pH 7.4). Dehydration was then achieved in a series of ethanol baths, and the samples were processed for embedding in a Spurr resin. Ultrathin sections were made using a Reichert-Young Ultracut microtome (Leica). Sections were contrasted with a 4% aqueous uranyl acetate solution and Reynold’s lead citrate before visualization. TEM imaging was performed with a Tecnai 12 operating at 80 kV equipped with a 1K×1K Keen View camera. For SEM, the samples were dried with a supercritical point dryer after ethanol baths.

### Statistical analysis

Data were analyzed by ANOVA and differences among means were compared by the Student–Newman–Keuls’ test using the general linear model by SAS version 9.1 (SAS Institute, Cary, NC, USA). Differences between different groups were considered statistically significant at P < 0.05. The results were presented as arithmetic mean ± standard error of mean (SEM).

## Abbreviations

ATP: Adenosine triphosphate; BB: Bold’s basal medium; CNF: Cellulose nanofibers; CNT: Carbon nanotube; EPS: Extracellular polymeric substance; Fv/Fm: Variable over maximal fluorescence; MWCNT: Multi-walled carbon nanotubes; NP: Nanoparticle; PAM: Pulsed amplitude modulation; PSII: *P*hotosystem II; PZC: Potential of zero charge; ROS: Reactive oxygen species; SEM: Scanning Electron Microscopy; SOD: Superoxide dismutase; SWCNT: Single-walled carbon nanotube; TEM: Transmission Electron Microscopy; XDR: X-ray diffraction; ZP: Zeta potential.

## Competing interests

The authors declare that they have no competing interests.

## Authors’ contributions

The work presented here was carried out in collaboration between all authors. CY, AC, NRBR, HMB and RB planned and supervised the series of experiments. MMP carried out the laboratory experiments and analyzed the data. MMP, NRBR and RB interpreted the results and wrote the paper. LM and JL performed the electronic microscopic analysis. JMM and LOL produced and characterized the nanoparticles. All of the authors approved the manuscript.

## Supplementary Material

Additional file 1: Table S1 Superoxide dismutase activity in *Chlorella vulgaris* exposed to Multi-walled carbon nanotubes (MWCNTs) or cotton cellulose nanofibers (CNFs) at different time points. Click here for file
