# Supplementary material for: Ecotoxicological effects of carbon nanotubes and cellulose nanofibers in Chlorella vulgaris
Source: J Nanobiotechnology. 2014 Apr 22;12:15. doi: 10.1186/1477-3155-12-15 (PMC4022149; doi:10.1186/1477-3155-12-15)
Supplement: Additional file 1: Table S1 — Superoxide dismutase activity in Chlorella vulgaris exposed to Multi-walled carbon nanotubes (MWCNTs) or cotton cellulose nanofibers (CNFs) at different time points. [file 1477-3155-12-15-S1.docx]

# Table S1

## Table 1 - Superoxide dismutase activity in Chlorella vulgaris exposed to Multi-walled carbon nanotubes (MWCNTs) or cotton cellulose nanofibers (CNFs) at different time points.

|  |  | Effect on medium | | | | | | | | | |
| --- | --- | --- | --- | --- | --- | --- | --- | --- | --- | --- | --- |
| Groups | Bold’s basal medium | | | | |  | | Seine river water | | | |
|  | 24 h | | 48 hrs | 72 hrs | 96 hrs |  | 24 hrs | | 48 hrs | 72 hrs | 96 hrs |
| Control | 33.29± 0.04^a^ | | 43.13± 0.03^a^ | 43.26± 0.03^a^ | 45.21± 0.03^a^ |  | 33.55± 0.02^a^ | | 37.31± 0.02^a^ | 39.38± 0.02^ab^ | 40.41± 0.02^b^ |
| 1 μg ml^-1^  MWCNT | 92.36± 0.01^d^ | | 85.49± 0.04^d^ | 70.60± 0.03^c^ | 68.65± 0.03^d^ |  | 92.36± 0.01^b^ | | 58.42± 0.07^b^ | 33.29 ±  0.19^a^ | 27.46± 0.04^a^ |
| 50 μg ml^-1^MWCNT | 94.95± 0.01^d^ | | 95.34± 0.01^e^ | 94.17± 0.01^e^ | 90.67± 0.02^e^ |  | 94.82± 0.01^b^ | | 60.10± 0.03^b^ | 58.68 ±  0.03^b^ | 40.67 ±  0.04 ^b^ |
| 100 μg ml^-1^MWCNT | 92.75± 0.03^d^ | | 94.17± 0.01^e^ | 94.30± 0.02^e^ | 91.97± 0.02^e^ |  | 94.56± 0.01^b^ | | 80.96± 0.15^c^ | 83.81 ±  0.21^c^ | 64.60± 0.20^d^ |
| 1 μg ml^-1^  CNF | 93.13± 0.01^d^ | | 81.22± 0.02^cd^ | 81.48± 0.02^d^ | 61.79± 0.02^c^ |  | 93.52± 0.01^b^ | | 64.25± 0.22^b^ | 51.30± 0.18^ab^ | 55.18± 0.14^cd^ |
| 50 μg ml^-1^  CNF | 62.56± 0.15^b^ | | 77.46± 0.04^c^ | 55.83± 0.03^b^ | 53.24± 0.06^b^ |  | 90.16 ±  0.02^b^ | | 37.31± 0.02^a^ | 33.94 ±  0.25^a^ | 46.37± 0.04^bc^ |
| 100 μg ml^-1^  CNF | 77.07± 0.06^c^ | | 65.93± 0.06^b^ | 58.81± 0.06^b^ | 48.32± 0.05^a^ |  | 67.62± 0.13^c^ | | 33.42± 0.08^a^ | 57.77 ±  0.03^b^ | 52.20± 0.07^bcd^ |

^a-f^ Means within the same column with different superscripts differ significantly at p<0.05. Data are presented as mean ± standard error from three independent experiments.
